# Temporal variation in associations between temperature and years of life lost in a southern China city with typical subtropical climate

**DOI:** 10.1038/s41598-017-04945-6

**Published:** 2017-07-05

**Authors:** Guoxing Li, Jing Huang, Guozhang Xu, Xiaochuan Pan, Xujun Qian, Jiaying Xu, Yan Zhao, Tao Zhang, Qichen Liu, Xinbiao Guo, Tianfeng He

**Affiliations:** 1 Department of Occupational and Environmental Health Sciences, Peking University School of Public Health, 38 Xueyuan Road, 100191 Beijing, China; 2 Ningbo Municipal Center for Disease Control and Prevention, 237 Yongfeng Road, 315010 Ningbo, China; 3 Ningbo First Hospital, Liuting Street, 315010 Ningbo, China; 4 Tulan University, 6823 St. Charles Avenue, New Orleans, LA 70118 USA

## Abstract

Though some studies have explored the association between temperature and years of life lost (YLL), limited evidence is available regarding the effect of temporal variation on the temperature-YLL relationship, especially in developing countries. We explored temporal variation in the associations between temperature and YLL before and after 2013 heat waves (period I: Jan 2008 to Sep 2013, period II: Oct 2013 to Dec 2015) in Ningbo, a southern China city with typical subtropical climate. The heat associations showed an increasing trend. The number of YLL due to heat-related respiratory mortality was significantly higher in period II (46.03, 95% CI: 11.97, 80.08) than in period I (7.21, 95% CI: −10.04, 24.46) among married individuals. In contrast, the cold associations presented an attenuating trend, and the number of YLL due to non-accidental mortality was significantly lower in period II (262.32, 95% CI: −304.18, 828.83) than in period I (916.78, 95% CI: 596.05, 1237.51). These results indicate more effort still needed to be made to reduce heat-related YLL even after periods of extreme heat. Furthermore, using YLL provided complementary information for identifying vulnerable subgroups, which has important implications for the planning of public health interventions.

## Introduction

Cold and hot weather are established risk factors for human health, and abundant epidemiologic evidence has indicated their association with excess risk of mortality and morbidity, especially due to cardiovascular and respiratory diseases^1–9^. Interest in this field has increased after episodes of extreme weather and under climate change scenarios^10^.

Although consensus regarding the adverse influence of cold and hot temperatures has been reached, these associations remain to be explored in further detail. For instance, uncertainty regarding future temperature-health relationships is acknowledged as one of the most important barriers to projecting health impact in climate change studies^11, 12^.

Consequently, temporal variation in the association between temperature and health needs to be explored in depth to gain an understanding of the manner in which changes in exposure levels and implementation of public health interventions influence this relationship, thus providing evidence for the prediction of future temperature-related health burden under climate change scenarios. Previous studies have assessed temporal changes in temperature-related mortality in the USA^13^, Italy^14, 15^, France^16^, Czech Republic^17^, etc.

In recent year, some studies have used years of life lost (YLL) as an index to estimate the relationship between temperature and disease burden^18–22^. YLL is a measure of disease burden that considers the life expectancy at death and, therefore, assigns higher weights to deaths that occur at younger ages^23^. This indicator has been extensively used to identify and prioritize causes of premature death around the world^24^. Evidence regarding the temperature-YLL relationship may provide additional information for policy making and health resource allocation. However, insights into the temporal variations in the temperature-YLL associations due to cause-specific mortality are limited, and the modifications of socioeconomic factors such, as marital status, are unclear.

Ningbo, located in the Yangtze River Delta in southern China, is the world’s fourth-largest port city. The population in Ningbo was estimated to be 7.83 million in 2015. Ningbo has a typical subtropical climate, with a hot summer and a mild winter. In 2013, the frequency of extremely hot days in the summer clearly exceeded that of other years in this southern Chinese city. The evaluation of temporal variation in the association between temperature and health before and after this period may have great importance for policy making regarding and the prediction of climate change in subtropical climate regions.

In order to provide evidence for the prediction of temperature-health associations and planning of public health interventions in subtropical climate regions under climate-change scenarios, we investigated temporal variation in associations between temperature and YLL due to cause-specific mortality in Ningbo, a southern Chinese city with typical subtropical climate during 2008–2015. In addition, the modifications of socioeconomic factors were also explored.

## Results

### Descriptive statistics

Table 1 shows the descriptive data for meteorological conditions, daily numbers of non-accidental, cardiovascular and respiratory deaths and their corresponding YLL in Ningbo during 2008 to 2015. The mean temperature was 17.6 °C, with a range from −2.2 °C to 34.4 °C identified during these years. Mean daily numbers of non-accidental, cardiovascular and respiratory deaths were 90.3, 27.7, and 15.4, respectively. The corresponding mean YLL were 1798.3, 459.0 and 203.7 for the three categories of diseases. The average daily YLL were higher in younger, male and married people than in elderly, female and widowed individuals, respectively.

**Table 1:** Descriptive statistics of meteorological factors, years of life lost and mortality from 2008 to 2015 in Ningbo, China.

**Variables**	**min**	**q25**	**median**	**q75**	**max**	**IQR**	**mean**	**SD**
**Daily meteorological measures**								
Temperature (°C)	−2.2	9.9	18.9	25.0	34.4	15.1	17.6	8.8
Relative humidity (%)	19.0	66.0	74.0	82.0	97.0	16.0	73.2	12.0
**Daily years of life lost (year)**								
Non-accidental	753.6	1569.9	1771.4	2004.1	3362.1	434.2	1798.3	323.3
Cardiovascular	128.5	361.8	447.0	540.3	1107.4	178.4	459.0	134.5
Respiratory	30.4	140.5	192.5	254.1	654.7	113.6	203.7	86.3
ge < 75	371.1	980.1	1131.8	1280.1	2348.6	300.0	1139.5	226.4
Age ≥ 75	271	535.6	631.9	763.8	1297.4	228.2	658.8	169.7
Male	429.1	946	1085.8	1243.1	2193.2	297.1	1100.8	223.8
Female	236.6	574.7	683.4	806.9	1369	232.2	697.5	171.9
Married	535.0	1093.3	1246.9	1407.7	2387.6	314.4	1256.2	231.8
Widowed	134.4	317.5	388.7	477.6	1028.8	160.1	404.8	119.5
**Daily deaths counts (No. of death)**								
Non-accidental	45	77	88	102	166	25	90.3	18.3
Cardiovascular	8	22	27	33	60	11	27.7	8.3
Respiratory	2	11	14	19	45	8	15.4	6.2
Age < 75	15	31	35	40	75	9	35.5	6.8
Age ≥ 75	22	44	52	64	108	20	54.8	14.7
Male	24	43	50	58	102	15	51.0	10.7
Female	15	32	38	46	79	14	39.3	10.0
Married	24	46	53	61	105	15	53.5	10.5
Widowed	11	26	31	39	76	13	32.8	9.8

### Overall cumulative associations

**Figure 1:**
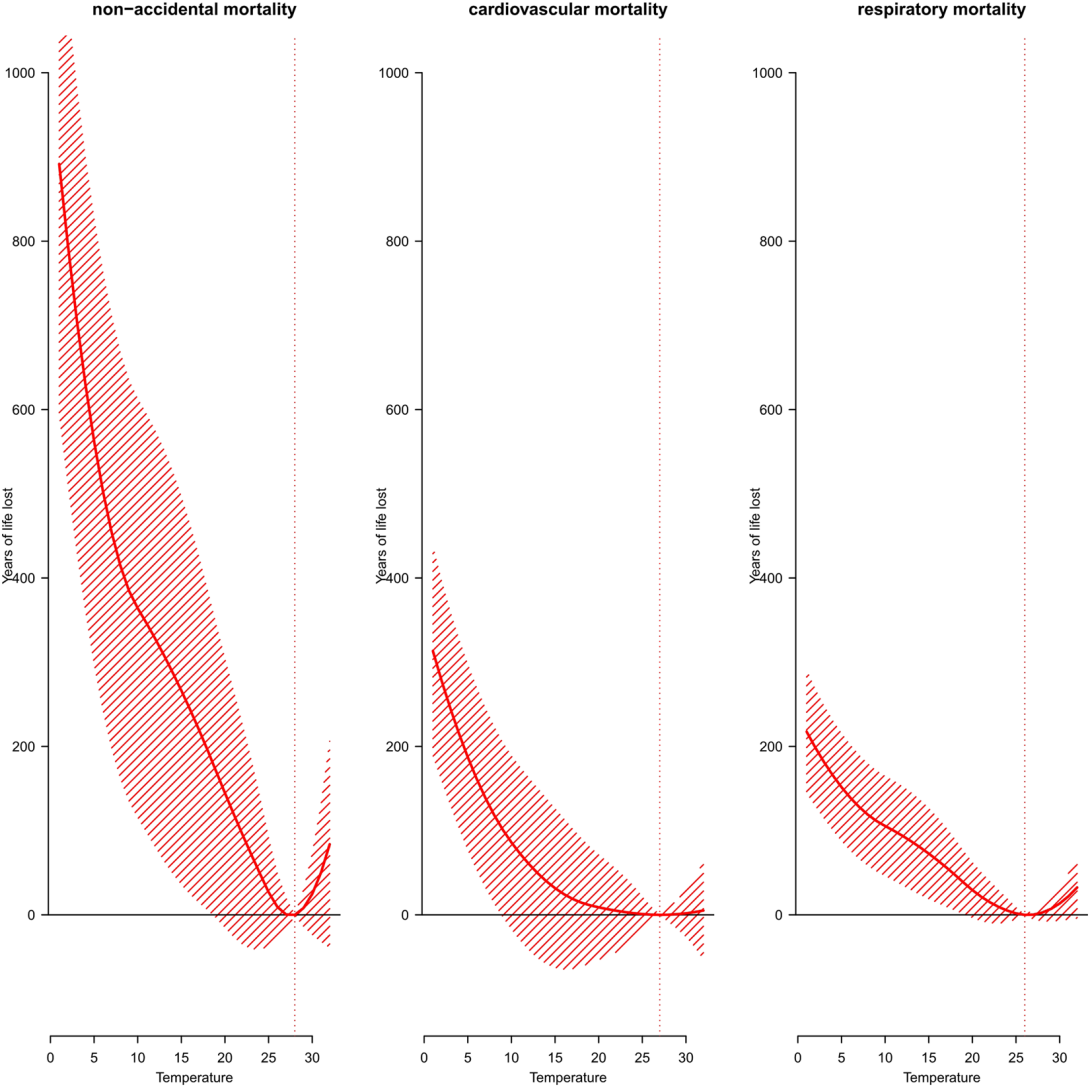
Overall cumulative exposure-response curves for the associations between temperature and years of life lost due to cause-specific mortality (non-accidental mortality, cardiovascular mortality, respiratory mortality) with 95% confidence intervals. The vertical lines represent the percentile of the minimum mortality temperature (dotted).

**Figure 2:**
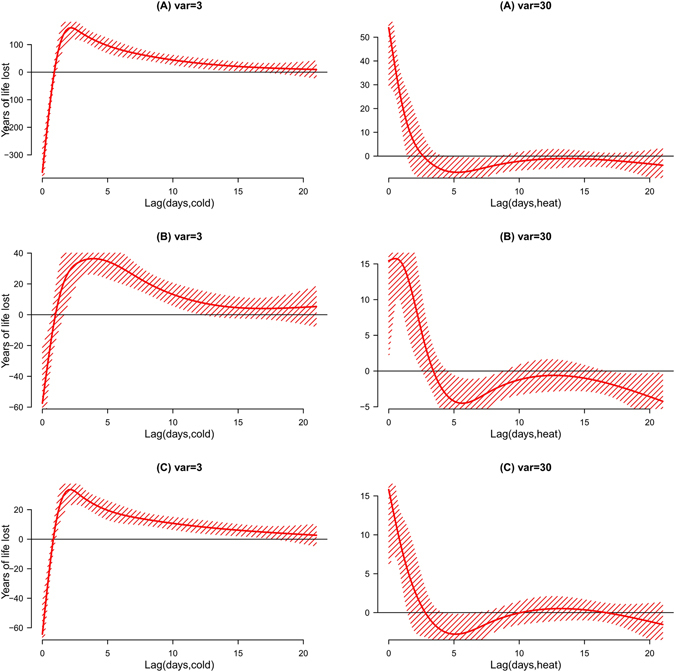
Lag-response curve for the association between heat and years of life lost due to cause-specific mortality ((**A**) non-accidental mortality, (**B**) cardiovascular mortality, (**C**) respiratory mortality), with 95% confidence intervals. These curves were computed based on temperatures corresponding to the 95^th^ & 5^th^ percentiles and compared with the minimum mortality temperature.

Figure 1 shows the exposure-response curves for the effects of daily mean temperature on YLL. In this figure, we detected the minimum mortality temperature (MMT), which indicated the temperature at which the lowest mortality risk occurs over the whole study period. When the temperature was higher or lower than MMP, mortality risk was expected to increase along with an increase or decrease of the temperature. Exploring MMT is important for policy making and health protection. All curves were U-shaped, with an MMT between 25 °C and 30 °C identified for non-accidental, cardiovascular and respiratory deaths. The lag effects of temperature on YLL showed that the cold effects peaked at 2 days post-exposure and declined slowly over the following 20 days, while the hot associations appeared acutely and declined rapidly over the following 2–3 days (Fig. 2). Therefore, we presented the cumulative hot and cold associations using a lag period of 21 days, which was sufficient to capture the lag associations of temperature.

The cumulative temperature-YLL associations due to non-accidental, cardiovascular, and respiratory mortality are shown in Table 2. In general, increased YLL were associated with high and low temperature exposure, and the cold associations were stronger than the hot associations.

**Table 2:** The cumulative cold and hot effects on years of life lost due to non-accidental, cardiovascular, and respiratory mortality.

Health Endpoints	Group	Period		Period	
		Jan 2008–Sep 2013 Cold: 5th vs MMP	Oct 2013–Dec 2015 Cold: 5th vs MMP	Jan 2008–Sep 2013 Hot: 95th vs MMP	Oct 2013–Dec 2015 Hot: 95th vs MMP
Non-accidental	ALL	916.78 (596.05, 1237.51)	262.32 (−304.18, 828.83)*	26.73 (−25.92, 79.37)	85.52 (−48.98, 220.03)
	Younger (<75)	332.24 (57.52, 606.96)	19.41 (−465.83, 504.65)	12.74 (−32.35, 57.83)	26.16 (−89.05, 141.37)
	Older (≥75)	584.53 (447.40, 721.66)	242.91 (0.70, 485.13)*	13.98 (−8.52, 36.49)	59.36 (1.85, 116.87)
	Male	476.05 (227.70, 724.41)	355.39 (−83.27, 794.06)	12.83 (−27.93, 53.60)	70.70 (−33.45, 174.85)
	Female	440.72 (252.29, 629.15)	−93.07 (−425.89, 239.76)*	13.89 (−17.04, 44.82)	14.82 (−64.20, 93.85)
	Married	504.75 (241.06, 768.43)	302.32 (−163.44, 768.08)	21.37 (−21.91, 64.65)	61.94 (−48.65, 172.52)
	Widowed	365.64 (255.846, 475.44)	−29.21 (−223.14, 164.73)*	13.73 (−4.30, 31.75)	21.64 (−24.41, 67.68)
Cardiovascular	ALL	290.60 (156.40, 424.80)	174.88 (−65.38, 415.14)	2.40 (−28.49, 33.29)	15.71 (−55.23, 86.64)
	Younger (<75)	94.02 (−13.06, 201.12)	77.43 (−114.29, 269.14)	−5.80 (−30.45, 18.85)	9.07 (−47.53, 65.67)
	Older (≥75)	196.58 (119.01, 274.15)	97.45 (−41.42, 236.33)	8.20 (−9.65, 26.05)	6.63 (−34.37, 47.63)
	Male	101.11 (0.27, 201.94)	54.76 (−125.76, 235.28)	−10.43 (−33.64, 12.78)	−7.90 (−61.20, 45.40)
	Female	189.49 (107.13, 271.86)	120.12 (−27.34, 267.57)	12.83 (−6.13, 31.78)	23.61 (−19.93, 67.14)
	Married	174.11 (63.64, 284.57)	106.69 (−91.06, 304.46)	−1.26 (−26.69, 24.16)	7.86 (−50.53, 66.24)
	Widowed	115.62 (53.76, 177.48)	23.80 (−86.95, 134.55)	3.21 (−11.03, 17.45)	−0.82 (−33.52, 31.88)
Respiratory	ALL	232.33 (154.21, 310.44)	80.74 (−59.87, 221.36)	10.83 (−12.52, 34.19)	59.76 (13.65, 105.88)
	Younger (<75)	27.61 (−23.48, 78.69)	22.69 (−69.26, 114.64)	−2.05 (−17.32, 13.22)	24.30 (−5.86, 54.45)
	Older (≥75)	204.72 (148.44, 261.00)	58.05 (−43.25, 159.36)*	12.88 (−3.94, 29.71)	35.47 (2.24, 68.69)
	Male	164.39 (106.38, 222.40)	38.86 (−65.57, 143.28)*	12.04 (−5.31, 29.38)	30.39 (−3.86, 64.63)
	Female	67.93 (18.88, 116.99)	41.89 (−46.41, 130.19)	−1.20 (−15.87, 13.46)	29.38 (0.42, 58.34)
	Married	102.28 (44.59,159.97)	61.65 (−42.20, 165.49)	7.21 (−10.04, 24.46)	46.03 (11.97, 80.08)*
	Widowed	132.90 (90.74, 175.05)	18.30 (−57.59, 94.18)*	9.10 (−3.50, 21.71)	21.21 (−3.67, 46.10)

MMP: minimum mortality percentile; The MMP for YLL of non-accidental, cardiovascular and respiratory mortality were 87, 83 and 79 percentiles, respectively. The 5th and 95th percentiles temperatures were 3.0 °C and 30.4 °C. *P < 0.05 (comparison between two periods).

### Temporal variation

**Figure 3:**
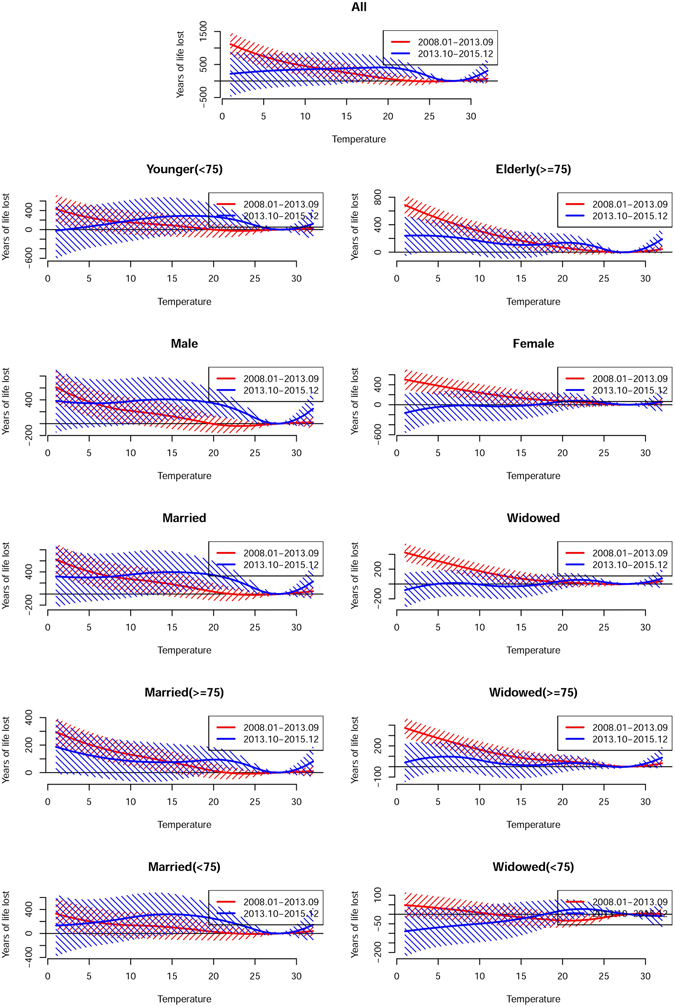
Overall cumulative exposure-response curves (with 95% confidence intervals) for the association between temperature and years of life lost due to non-accidental mortality during period I (Jan 2008 to Sep 2013, red) and period II (Oct 2013 to Dec 2015, blue).

**Figure 4:**
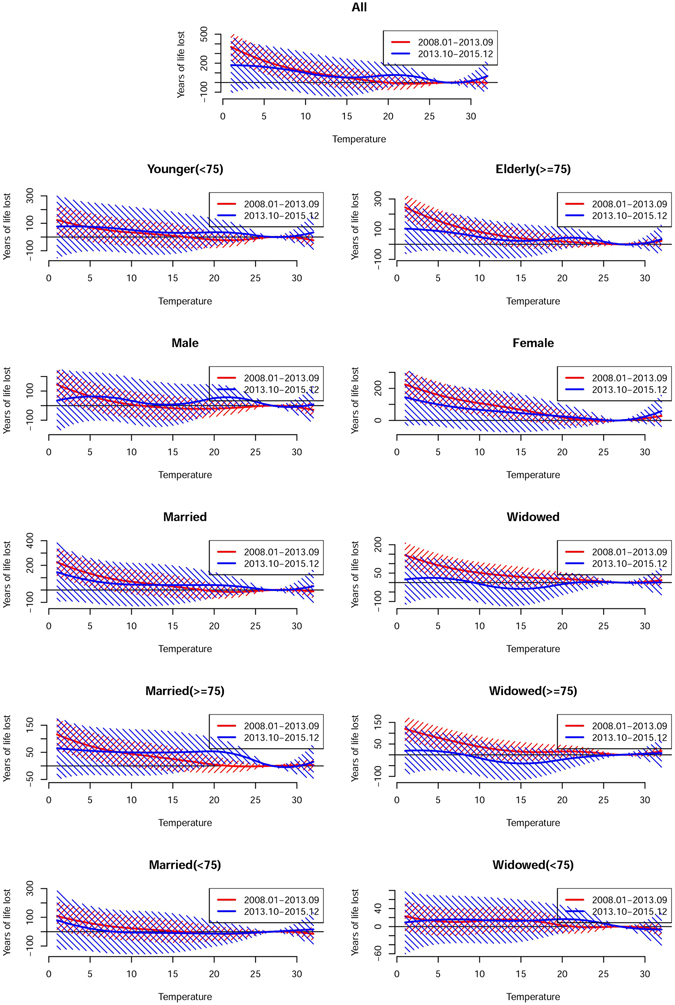
Overall cumulative exposure-response curves (with 95% confidence intervals) for the association between temperature and years of life lost due to cardiovascular mortality during period I (Jan 2008 to Sep 2013) (red) and period II (Oct 2013 to Dec 2015) (blue).

**Figure 5:**
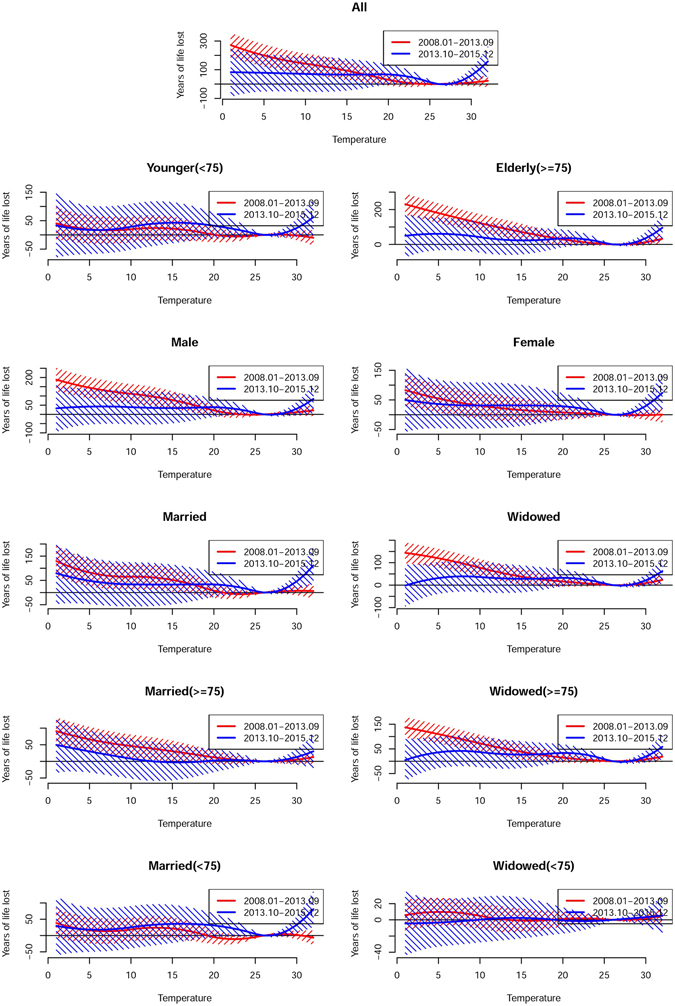
Overall cumulative exposure-response curves (with 95% confidence intervals) for the association between temperature and years of life lost due to respiratory mortality during period I (Jan 2008 to Sep 2013) (red) and period II (Oct 2013 to Dec 2015) (blue).

The major results of the analysis of temporal variation in the association between temperature and YLL are summarized in Table 2 and Figs 3–5 (Fig. 3 for non-accidental disease, Fig. 4 for cardiovascular disease, Fig. 5 for respiratory disease). The figures show the exposure-response curves predicted based on the time-varying distributed lag non-linear model (DLNM) for period I (Jan 2008 to Sep 2013, Red Line) and period II (Oct 2013 to Dec 2015, Blue Line).

In general, the analysis suggested that the trend in heat-related YLL was steeper in period II than period I. When stratified by modifying factors, the YLL due to heat-related respiratory mortality were significantly higher in period II (46.03, 95% CI: 11.97, 80.08) years than in period I (7.21, 95% CI: −10.04, 24.46) years in married individuals. In contrast, the cold associations presented an attenuating trend, and the YLL due to non-accidental mortality was significantly lower in period II (262.32, 95% CI: −304.18, 828.83) years than in period I (916.78, 95% CI: 596.05, 1237.51) years. In the stratification analysis, YLL also showed a significant decrease, and the YLL due to non-accidental and respiratory mortality was significantly lower in the elderly and widowed individuals in period II than period I. In addition, the analysis of the curves suggested that more pronounced variations were associated with extreme temperatures (Figs 3–5).

The estimates for the temporal variation in the temperature-mortality association are presented in Table 3. Similar trends were identified; in particular, respiratory mortality exhibited the greatest relative increase in association with the effect of heat, and non-accidental mortality presented the greatest relative decrease in cold associations, with relative risk (RR) at the 95^th^ temperature percentile increasing from 1.08 (95% CI: 0.97, 1.20) in period I to 1.44 (95% CI:1.16, 1.80) in period II, and RR at the 5^th^ temperature percentile dropping from 1.90 (95% CI:1.62, 2.22) in period I to 1.34 (95% CI: 1.02, 1.77) in period II.

**Table 3:** The cumulative cold and hot effects on mortality risk due to non-accidental, cardiovascular, and respiratory mortality.

Health Endpoints	Group	Period		Period	
		Jan 2008–Sep 2013 Cold: 5th vs MMP	Oct 2013–Dec 2015 Cold: 5th vs MMP	Jan 2008–Sep 2013 Hot: 95th vs MMP	Oct 2013–Dec 2015 Hot: 95th vs MMP
Non-accidental mortality	ALL	1.90 (1.62, 2.22)	1.34 (1.02, 1.77)*	1.03 (0.99, 1.07)	1.08 (0.99, 1.17)
	Younger ( < 75)	1.39 (1.11, 1.73)	1.08 (0.72, 1.61)	1.03 (0.98, 1.09)	1.02 (0.90, 1.15)
	Older ( ≥ 75)	2.34 (1.92, 2.85)	1.51 (1.07, 2.13)*	1.02 (0.97, 1.07)	1.12 (1.01, 1.25)
	Male	1.78 (1.46, 2.17)	1.48 (1.05, 2.10)	1.03 (0.98, 1.08)	1.05 (0.95, 1.17)
	Female	2.06 (1.65, 2.58)	1.17 (0.79, 1.74)*	1.03 (0.98, 1.09)	1.11 (0.98, 1.26)
	Married	1.67 (1.38, 2.03)	1.42 (1.02, 1.98)	1.03 (0.99, 1.08)	1.05 (0.95, 1.16)
	Widowed	2.36 (1.85, 3.02)	1.22 (0.79, 1.89)*	1.03 (0.97, 1.09)	1.10 (0.96, 1.27)
Cardiovascular mortality	ALL	1.86 (1.43,2.42)	1.45 (0.93, 2.27)	1.03 (0.97, 1.10)	1.04 (0.90, 1.19)
	Younger (<75)	1.59 (0.99, 2.55)	1.44 (0.60, 3.46)	0.98 (0.88, 1.10)	1.01 (0.77, 1.32)
	Older (≥75)	1.98 (1.45, 2.70)	1.46 (0.87, 2.42)	1.05 (0.97, 1.13)	1.04 (0.89, 1.22)
	Male	1.52 (1.07, 2.16)	1.41 (0.77, 2.57)	0.99 (0.90, 1.08)	0.96 (0.80, 1.16)
	Female	2.29 (1.59, 3.31)	1.47 (0.79, 2.72)	1.08 (0.98, 1.18)	1.12 (0.92, 1.36)
	Married	1.81 (1.27, 2.57)	1.49 (0.82, 2.71)	1.02 (0.93, 1.11)	1.02 (0.85, 1.23)
	Widowed	1.98 (1.33, 2.93)	1.27 (0.65, 2.47)	1.04 (0.94, 1.14)	1.02 (0.83, 1.26)
Respiratory mortality	ALL	2.82 (2.03, 3.93)	1.76 (0.95, 3.26)	1.08 (0.97, 1.20)	1.44 (1.16, 1.80)*
	Younger (<75)	1.53 (0.63, 3.74)	2.07 (0.36, 12.00)	1.01 (0.75, 1.35)	1.90 (1.02, 3.54)
	Older (≥75)	3.09 (2.18, 4.39)	1.74 (0.91, 3.327)	1.09 (0.97, 1.22)	1.39 (1.10, 1.76)
	Male	3.94 (2.51, 6.19)	1.58 (0.69, 3.62)	1.09 (0.94, 1.26)	1.32 (0.98, 1.77)
	Female	2.00 (1.26, 3.16)	1.96 (0.82, 4.69)	1.07 (0.91, 1.25)	1.61 (1.17, 2.21)*
	Married	2.48 (1.51, 4.07)	2.05 (0.83, 5.03)	1.03 (0.88, 1.22)	1.44 (1.05, 1.98)
	Widowed	3.29 (2.13, 5.10)	1.71 (0.74, 3.95)	1.13 (0.98, 1.31)	1.45 (1.07, 1.97)

MMP: minimum mortality percentile; The MMP for RR of non-accidental, cardiovascular and respiratory mortality were 83, 83 and 79 percentiles, respectively. The 5th and 95th percentiles temperatures were 3.0 °C and 30.4 °C. *P < 0.05 (comparison between two periods).

### Subgroup comparisons

In general, the elderly tended to have higher YLL associated with both hot and cold temperatures than did younger individuals. However, significant differences were only observed in cold-related respiratory disease during period I, with an increase of 204.72 (95% CI: 148.44, 261.00) years in YLL observed in the elderly and a change of 27.61 (95% CI: −23.48, 78.69) years in YLL observed in younger individuals. When gender was considered as a modifying factor, a higher number of YLL of cold-related respiratory deaths were observed in males relative to females during period I. For marital status, the results showed that YLL were higher among married than widowed individuals, while a significant difference was only detected in heat-related respiratory disease during period II, with an increase of 46.03 (95% CI: 11.97, 80.08) years in YLL observed in married individuals and a change of 21.21 (95% CI: −3.67, 46.10) years in YLL observed in widowed individuals. When stratified marital status by age, YLL were also higher among married than widowed people. For instance, an increase of 32.88 (95% CI: 8.59, 57.17) years and a change of 3.39 (95% CI: −7.34, 14.21) years in YLL observed in married and widowed people who were less than 75 years old, respectively. The detailed results were presented in Table S5.

When stratified by age and gender, the temperature-mortality association and the temperature-YLL relationship showed the same trend. However, when modified by marital status, they were different (Table 3). Widowed individuals tended to be at higher risk than married individuals. A significant difference was detected in cold-related respiratory disease during period I, with increased risks of 2.36 (95% CI: 1.85, 3.02) and 1.67 (95% CI: 1.38, 2.03) observed in widowed and married individuals, respectively. When considering age in different marital status, similar results were shown. For example, increased risks of 2.52 (95% CI: 1.95, 3.26) and 2.11 (95% CI: 1.59, 2.79) detected in married and widowed people older than 75 years, respectively. More details were presented in Table S6.

### Sensitivity analyses results

Sensitivity analyses were performed to test whether the results were robust based on the variation of the parameters in the model. The supplemental material shows that the estimations were stable when extending the maximum lag period to 27 days (see Supplementary Fig. S1), using 6 or 8 degrees of freedom per year for the time variable (see Supplementary Figs S2 and S3), removing relative humidity from the analysis (see Supplementary Fig. S4).

## Discussion

The study found hot and cold temperatures had significant influences on YLL due to non-accidental, cardiovascular and respiratory mortality in general. In addition, the results indicated that an increase in YLL was associated with heat, while an attenuating trend was observed in association with cold after an extreme hot period in a southern Chinese city with typical subtropical climate.

The hot associations were acute and lasted for 2–3 days, while the cold associations were delayed and lasted for approximately 20 days. This evidence may provide information for public health and other relevant departments to develop early response plans for cold and hot temperatures. Furthermore, the results of our study showed that the associations of cold were stronger than the associations of heat, which was consistent with previous studies^19, 25^. Ningbo is a southern Chinese city with a typical subtropical climate, and the annual mean temperature is relatively high. The residents in this subtropical region may have stronger physiological and behavioural adaptions to high temperatures, while their adaptive capacity to cold is relatively weak. A previous study also showed that the effect of cold in warmer cities was increased relative to that in cold cities^26^. This information also indicated that the adverse influence of low temperature should not be ignored in subtropical regions, even under the global warming scenario.

The study identified an increasing YLL trend after the extremely hot period, although the mean (26.5 °C) and maximum summer temperatures (31.9 °C) were lower in period II when compared with the mean (28.0 °C) and maximum summer temperatures (33.3 °C) in period I (Supplementary Table S1). In addition, this result was discordant with the findings of previous studies, which indicated that heat-related associations decreased after the specific weather events^15–17^.

Changes in temperature-related associations over a long period of time may be explained by infrastructural changes, such as improvements in housing and health service and physiological acclimatization^27, 28^. However, over a relatively short period of time, any changes in infrastructure and physiological acclimatization would likely be small. Studies comparing the association between heat and mortality before and after the 2003 heat waves in Europe indicated that the implementation of public health interventions may have played an important role in reducing the risk^15–17^.

To our knowledge, many interventions were implemented during the extremely hot period in Ningbo in 2013. These interventions included the issuance of early warning information by the meteorological and health departments, increased heat stroke surveillance, and restriction of outdoor work during the extreme hot period. The results indicated these interventions were effective at reducing health risks during this extreme hot period. This may be because, beyond the effects of the intervention programmes itself, such interventions may increase the level of awareness regarding the health risks associated with extreme temperatures, promote behavioural adaptation, etc. However, after the extremely hot period, the mean and maximum temperatures decreased, and it was possible that decreased attention was paid to temperature-related health risks, reducing the effectiveness of the interventions; therefore, the health risks associated with hot temperature increased during this period.

Contrary to the increased YLL observed in association with the heat associations, temporal variations in the cold associations exhibited an attenuating trend. The mean and minimum winter temperatures were 11.2 °C and 0.6 °C, respectively, in period II, which were higher than the mean (10.5 °C) and minimum winter temperatures (−1.1 °C) in period I (see Supplementary Table S1). The lower impact of cold observed during the latter period relative to the former period may be partly explained by the mild winter temperatures observed during the latter period. The results regarding the aforementioned temporal variations may have important implications for the prediction of and policy making related to climate change in subtropical climate regions.

In this study, we explored the modifications of marital status on the temperature-health relationship using YLL as an indicator. We found widowed individuals had a higher risk of mortality than did married people. However, when using YLL as an indicator, the results showed a different pattern, with a higher YLL identified in married persons.

Consistent with previous studies^29, 30^, our study also found that elderly individuals were at higher risk of mortality than were younger individuals in association with both cold and hot temperatures. When using YLL as an indicator, elderly individuals also tended to have higher YLL than did younger individuals. Recently, researchers in Chongqing, Guangzhou and Zhuhai in China have also shown that the associations of high and low temperatures on YLL were higher in people aged ≥ 65 years than those aged <65 years^21, 22^. This finding was plausible from a biological perspective. Because thermal regulation systems may weaken with age, sensory perceptions may diminish and thermal homeostasis may decline^31^. In addition, elderly individuals frequently have pre-existing chronic diseases, which may make them more vulnerable to the adverse influence of temperature.

Gender also served as a modifying factor in the temperature-health association. A significant higher number of YLL and increased mortality risk due to respiratory disease were found in males. The difference may due to the socioeconomic and biological differences between the two genders.

Compared with mortality risk, using YLL as an indicator takes the age at which death occurs into account and, therefore, is useful for estimating premature death, which indicates the preventable years of life lost. Based on Table 1, we can see that although the number of married individuals was approximately 1.70 times the number of widowed individuals, the YLL of the former was 3.21 times that of the latter, which means the former had more preventable years of life lost. Taking the occurrence of death at different ages into consideration may result in more efficient resource allocation. The results of our study indicated that although widowed individuals were susceptible to extreme temperatures possibly due to the psychological vulnerability associated with spouse loss and living alone^32^, married people should not be ignored when implementing efforts to combat the harmful influence of extreme temperatures.

Our study has several strengths. First, we explored temporal variation in the associations of temperature on health before and after heat waves using the indicator of YLL. Second, the use of time-varying DLNM could assess exposure-response associations as continuous, non-linear shapes and account for the associations that cumulated over a lag period, thus generate a better understanding of the temperature-health associations and provide evidence for the future predictions. Third, researches analysing the modification of marital status on the temperature-YLL relationship were limited.

However, the data used in this study were only from one city in a subtropical region, and cautions should be taken when generalizing the results to other geographic areas. A large, multi-city study in a developing country might be higher impact in terms of adding to the literature on temperature and health. Second, temperature data were collected from a fixed monitoring site rather than indoors, but measurement errors may bias the results towards the null hypothesis. Third, the confounding effect of air pollutants was not controlled, but previous studies have reported that the associations of temperature were robust even after controlling for air pollutants^33^. Improvements need to be made in the future studies.

As a conclusion, our study provided insights into temporal variations in the temperature-YLL associations in a southern China city with typical subtropical climate. The heat associations increased after the extreme hot period, while the cold associations attenuated after this period. The temporal variation results indicated that there is a continued need for more efforts to be made to reduce heat-related YLL, even after periods of extreme heat have ended. In addition, the use of YLL as a mortality indicator provided a complementary method for detecting vulnerable subgroups, which may have important implications for the planning of public health interventions.

## Methods

### Data of mortality and YLL

The study was approved by the Institutional Review Board of Ningbo Municipal Center for Disease Control and Prevention (IRB 201603). Mortality data, including the underlying cause of death, were obtained from Ningbo Municipal Center for Disease Control and Prevention between January 2008 and December 2015, and a total of 263,789 registered non-accidental deaths were identified. Causes of death were classified using the International Classification of Diseases 10th version (ICD-10), and deaths due to non-accidental (A00-R99) and cardiovascular (I00-99) and respiratory (J00-99) diseases were analysed. The dataset comprised date of death, sex, age and marital status. Daily death count was defined as the number of deaths occurring on a single day. We calculated YLL by matching the patient’s age to the life table for each death. The World Health Organization (WHO) standard life table for YLL was used (see Supplementary Table S2). Daily YLL were calculated by summing the YLL for all deaths on the same day. We stratified the sums by age (<75 and ≥75 years), gender (male and female) and marital status (the married and the widowed). Considering marital status, the number of married and widowed people accounted for 95% of the total number of deaths, thus we considered these two groups in the analysis.

### Data of weather conditions

Daily meteorological data, including temperature and relative humidity, were obtained from the local meteorological bureau.

### Data analysis

Generalized linear model (GLM) with a quasi-Poisson family was applied to derive the exposure-response curve for the association between temperature and mortality^34^. We included a variable for calendar days with a natural cubic spline function (7 degrees per year) to adjust for confounding related to seasonality and short-term fluctuations using day of the week as a factor. A natural cubic spline with 3 degrees of freedom was used for the daily relative humidity variable. To effectively measure for the effect of temperature, a distributed lag non-linear model (DLNM) was used^35^. This model can combine the conventional exposure-response association and the additional lag-response to describe complex non-linear and lagged dependencies, respectively. Specifically, we selected a cross-basis composed of a quadratic β-spline for the exposure-response function with three internal knots placed at equally spaced temperature percentiles (25^th^, 50^th^ and 75^th^) and a natural cubic β-spline for the lag-response function with an intercept and three internal knots placed at equally spaced values on the log scale. Previous studies have shown that the lag effect of cold temperature persists for nearly two weeks, while the lag effect of hot temperature persists for less than one week; therefore, we chose a lag period of 21 days, which was sufficient to capture the effect of temperature. We tested these modelling approaches using sensitivity analyses. We validated the fit of the model by checking the residuals to ensure that autocorrelation had been successfully removed.

We determined the minimum mortality temperature (MMT) based on the exposure-response curves for cause-specific mortality. We estimated the relationship between daily YLL and temperature to facilitate a comparison between YLL and mortality. Because daily YLL had a normal distribution (see Supplementary Fig. S5), we used the Gaussian family. The independent variables that were used in the mortality model were also used in the YLL model. To ease interpretation, the curves were rescaled by centring them on the MMT derived based on cause-specific mortality and YLL.

In 2013, the frequency of extreme temperature days in the summer clearly exceeded that of other years (see Supplementary Table S3). To assess temporal variation in the temperature-YLL and temperature-mortality associations before and after that summer, we extended the first-stage models by adding a linear interaction between time and the cross-basis variables^36^. A parameterization index was derived by directly defining the main and interaction terms in the model. The former were the cross-basis variables described during the first stage of analysis, while the latter were interaction terms created by multiplying the main terms with a dummy variable (0 or 1) for time period. Regarding the lag effects of temperature, we selected Sep 30, 2013 as the cut point, which means that the time interval before the extreme hot period was considered to be Jan 2008 to Sep 2013 (period I) and the time interval after the extremely hot period was considered to be Oct 2013 to Dec 2015 (period II). Using time-varying DLNM, we predicted the exposure-lag-response association between the two periods. In addition, we stratified the analyses by age, gender and marital status. Considering age is strongly correlated with marital status, we stratified the marital status by age, and the findings on marital status were re-tested with adjustment for age. Significance tests of differences in the two periods were based on the coefficient of the interaction terms^25^.

### Sensitivity analyses

Sensitivity analyses were performed on the parameters included in the models to test the robustness of our results. We extended the temperature lag to 27 days. We also plotted the extreme temperature exposure-lag-response curve to test whether the effect of temperature on health outcomes was effectively controlled. We also modified the degrees of freedom for time (6 & 8df). We removed relative humidity from these analyses to test the robustness of the results.

In our analysis, the *dlnm* package in R software (version 3.1.2) was used to perform all analyses. *P* < 0.05 (2 sided) were considered to be significant.

## Electronic supplementary material

Supplemental material
