# Supplementary material for: Temporal variation in associations between temperature and years of life lost in a southern China city with typical subtropical climate
Source: Sci Rep. 2017 Jul 5;7:4650. doi: 10.1038/s41598-017-04945-6 (PMC5498602; doi:10.1038/s41598-017-04945-6)
Supplement: Supplementary file 1 — Supplemental material [file 41598_2017_4945_MOESM1_ESM.doc]

**Supplemental Material**

**Temporal variation in associations between temperature and years of life lost in a southern China city with typical subtropical climate**

Guoxing Li^1a^, Jing Huang^1a^, Guozhang Xu^2^, Xiaochuan Pan^1^, Xujun Qian^3^, Jiaying Xu^4^, Yan Zhao^1^, Tao Zhang^2^, Qichen Liu^1^, Xinbiao Guo^1*^, Tianfeng He^2*^

^a^ These two authors contributed equally to this study.

*Corresponding authors

^1^ Department of Occupational and Environmental Health Sciences, Peking University School of Public Health, 38 Xueyuan Road, 100191, Beijing, China

^2^ Ningbo Municipal Center for Disease Control and Prevention, 237 Yongfeng Road, 315010, Ningbo, China

^3^ Ningbo First Hospital, Liuting Street, 315010, Ningbo, China

^4^ Tulan University, 6823 St. Charles Avenue New Orleans, LA 70118, USA

*Correspondence to:

Xinbiao Guo, MD, PhD

Department of Occupational and Environmental Health Sciences

Peking University School of Public Health

38 Xueyuan Road, Beijing 100191, China

E-mail: [guoxb@bjmu.edu.cn](mailto:guoxb@bjmu.edu.cn)

Tianfeng He, Chief Physician

Ningbo Municipal Center for Disease Control and Prevention,

Haishu District, 237 Yongfeng Road, Ningbo 315010, China

E-mail: [hetianfeng82@163.com](mailto:hetianfeng82@163.com)

**Tables:**

Table S1. Temperature variations in summer and winter during 2008 to 2015 in Ningbo, China

Table S2. WHO Standard life table for years of life lost

Table S3 The frequency of extreme temperature days in summer during 2008 to 2015 in Ningbo, China

Table S4. Descriptive statistics of years of life lost and mortality in the married and widowed people from 2008 to 2015 in Ningbo, China

Table S5. The cumulative cold and hot effects on years of life lost in the married and widowed people from 2008 to 2015 in Ningbo, China

Table S6. The cumulative cold and hot effects on mortality risk in the married and widowed people from 2008 to 2015 in Ningbo, China

**Figures:**

Fig.S1. Overall cumulative exposure-response curves (with 95% confidence intervals) for the association between temperature and YLL due to non-accidental, cardiovascular and respiratory mortality during period I (Jan 2008 to Sep 2013, red) and period II (Oct 2013 to Dec 2015, blue) when extending the maximum lag period to 27 days.

Fig.S2. Overall cumulative exposure-response curves (with 95% confidence intervals) for the association between temperature and YLL due to non-accidental, cardiovascular and respiratory mortality during period I (Jan 2008 to Sep 2013, red) and period II (Oct 2013 to Dec 2015, blue) when using 6 degrees of freedom per year for the time variable.

Fig.S3. Overall cumulative exposure-response curves (with 95% confidence intervals) for the association between temperature and YLL due to non-accidental, cardiovascular and respiratory mortality during period I (Jan 2008 to Sep 2013, red) and period II (Oct 2013 to Dec 2015, blue) when using 8 degrees of freedom per year for the time variable.

Fig.S4. Overall cumulative exposure-response curves (with 95% confidence intervals) for the association between temperature and YLL due to non-accidental, cardiovascular and respiratory mortality during period I (Jan 2008 to Sep 2013, red) and period II (Oct 2013 to Dec 2015, blue) when removing relative humidity from the analysis.

Fig.S5. Distribution of daily YLL for non-accidental, cardiovascular and respiratory mortality.

**Table S1. Temperature variations in summer and winter during 2008 to 2015 in Ningbo, China**

| Year | Summer(June, July, August) | | Winter(December, January, February) | |
| --- | --- | --- | --- | --- |
|  | mean | maximum | mean | minimum |
| 2008 | 27.7 | 32.2 | 10.3 | -0.3 |
| 2009 | 27.9 | 33.9 | 10.9 | -2.2 |
| 2010 | 27.6 | 33.8 | 11.1 | -0.6 |
| 2011 | 27.9 | 32.6 | 10.3 | -1.6 |
| 2012 | 28.0 | 33 | 9.9 | -0.7 |
| 2013 | 29.0 | 34.4 | 10.6 | -0.8 |
| 2014 | 26.5 | 31.9 | 11.4 | 0.6 |
| 2015 | 26.5 | 31.9 | 11.7 | 2.1 |

**Table S2. WHO Standard life table for years of life lost**

|  | Age | | SEYLL* | | Age | | SEYLL | | Age | | SEYLL | |  | |
| --- | --- | --- | --- | --- | --- | --- | --- | --- | --- | --- | --- | --- | --- | --- |
|  | 0 | | 91.94 | | 35 | | 57.15 | | 70 | | 23.15 | |  | |
|  | 1 | | 91 | | 36 | | 56.16 | | 71 | | 22.23 | |  | |
|  | 2 | | 90.01 | | 37 | | 55.17 | | 72 | | 21.31 | |  | |
|  | 3 | | 89.01 | | 38 | | 54.18 | | 73 | | 20.4 | |  | |
|  | 4 | | 88.02 | | 39 | | 53.19 | | 74 | | 19.51 | |  | |
|  | 5 | | 87.02 | | 40 | | 52.2 | | 75 | | 18.62 | |  | |
|  | 6 | | 86.02 | | 41 | | 51.21 | | 76 | | 17.75 | |  | |
|  | 7 | | 85.02 | | 42 | | 50.22 | | 77 | | 16.89 | |  | |
|  | 8 | | 84.02 | | 43 | | 49.24 | | 78 | | 16.05 | |  | |
|  | 9 | | 83.03 | | 44 | | 48.25 | | 79 | | 15.22 | |  | |
|  | 10 | | 82.03 | | 45 | | 47.27 | | 80 | | 14.41 | |  | |
|  | 11 | | 81.03 | | 46 | | 46.28 | | 81 | | 13.63 | |  | |
|  | 12 | | 80.03 | | 47 | | 45.3 | | 82 | | 12.86 | |  | |
|  | 13 | | 79.03 | | 48 | | 44.32 | | 83 | | 12.11 | |  | |
|  | 14 | | 78.04 | | 49 | | 43.34 | | 84 | | 11.39 | |  | |
|  | 15 | | 77.04 | | 50 | | 42.36 | | 85 | | 10.7 | |  | |
|  | 16 | | 76.04 | | 51 | | 41.38 | | 86 | | 10.03 | |  | |
|  | 17 | | 75.04 | | 52 | | 40.41 | | 87 | | 9.38 | |  | |
|  | 18 | | 74.05 | | 53 | | 39.43 | | 88 | | 8.76 | |  | |
|  | 19 | | 73.05 | | 54 | | 38.46 | | 89 | | 8.16 | |  | |
|  | 20 | | 72.06 | | 55 | | 37.49 | | 90 | | 7.6 | |  | |
|  | 21 | | 71.06 | | 56 | | 36.52 | | 91 | | 7.06 | |  | |
|  | 22 | | 70.07 | | 57 | | 35.55 | | 92 | | 6.55 | |  | |
|  | 23 | | 69.07 | | 58 | | 34.58 | | 93 | | 6.07 | |  | |
|  | 24 | | 68.08 | | 59 | | 33.62 | | 94 | | 5.6 | |  | |
|  | 25 | | 67.08 | | 60 | | 32.65 | | 95 | | 5.13 | |  | |
|  | 26 | | 66.09 | | 61 | | 31.69 | | 96 | | 4.65 | |  | |
|  | 27 | | 65.09 | | 62 | | 30.73 | | 97 | | 4.18 | |  | |
|  | 28 | | 64.1 | | 63 | | 29.77 | | 98 | | 3.7 | |  | |
|  | 29 | | 63.11 | | 64 | | 28.82 | | 99 | | 3.24 | |  | |
|  | 30 | | 62.11 | | 65 | | 27.86 | | 100 | | 2.79 | |  | |
|  | 31 | | 61.12 | | 66 | | 26.91 | | 101 | | 2.36 | |  | |
|  | 32 | | 60.13 | | 67 | | 25.96 | | 102 | | 1.94 | |  | |
|  | 33 | | 59.13 | | 68 | | 25.02 | | 103 | | 1.59 | |  | |
|  | 34 | | 58.14 | | 69 | | 24.08 | | 104 | | 1.28 | |  | |
|  |  | |  | |  | |  | | 105 | | 1.02 | |  | |
|  | *SEYLL: standard expected years of life lost. (from Global Health Estimates Technical Paper WHO/HIS/HSI/ GHE/ 2013.4)  **Table S3 The frequency of extreme temperature days in summer during 2008 to 2015 in Ningbo, China** | | | | | | | | | | | |  | |
| Percentiles | | 2008 | 2009 | 2010 | | 2011 | | 2012 | | 2013 | | 2014 | | 2015 |
| 90% | | 32 | 27 | 41 | | 27 | | 35 | | 52 | | 16 | | 15 |
| 95% | | 13 | 12 | 15 | | 10 | | 13 | | 37 | | 3 | | 9 |
| 98% | | 3 | 2 | 7 | | 1 | | 5 | | 20 | | 0 | | 0 |

**Table S4. Descriptive statistics of years of life lost and mortality in the married and widowed people** from 2008 to 2015 in Ningbo, China

| **Variables** | | **min** | **q25** | **median** | **q75** | **max** | **IQR** | **mean** | **SD** |
| --- | --- | --- | --- | --- | --- | --- | --- | --- | --- |
| **Years of life lost(years)** | | | | | | | | | |
| Married | 535.0 | | 1093.3 | 1246.9 | 1407.7 | 2387.6 | 314.4 | 1256.2 | 231.8 |
| <75 | 290.4 | | 799.5 | 928.4 | 1053.5 | 1927.3 | 254.0 | 932.7 | 193.4 |
| ≥75 | 76.8 | | 255.8 | 314.9 | 382.2 | 704.6 | 126.3 | 323.5 | 94.7 |
| Widowed | 134.4 | | 317.5 | 388.7 | 477.6 | 1028.8 | 160.1 | 404.8 | 119.5 |
| <75 | 0 | | 49.2 | 79.1 | 115.1 | 329.6 | 65.9 | 86.4 | 48.0 |
| ≥75 | 76.8 | | 255.8 | 314.9 | 382.2 | 704.5 | 126.3 | 323.5 | 94.7 |
| **Daily death counts(No. of deaths)** |  | |  |  |  |  |  |  |  |
| Married | 24 | | 46 | 53 | 61 | 105 | 15 | 53.5 | 10.5 |
| <75 | 11 | | 25 | 29 | 33 | 59 | 8 | 29.3 | 6.0 |
| ≥75 | 6 | | 19 | 23 | 29 | 56 | 10 | 24.2 | 7.3 |
| Widowed | 11 | | 26 | 31 | 39 | 76 | 13 | 32.8 | 9.8 |
| <75 | 0 | | 2 | 3 | 4 | 12 | 2 | 3.4 | 1.9 |
| ≥75 | 9 | | 23 | 28 | 35 | 68 | 12 | 29.5 | 9.2 |

**Table S5. The cumulative cold and hot effects on years of life lost in the married and widowed people from 2008 to 2015 in Ningbo, China**

|  |  | Period | | Period | |
| --- | --- | --- | --- | --- | --- |
| Health Endpoints | Group | Jan 2008-Sep 2013  Cold:5th vs MMP | Oct 2013-Dec 2015  Cold:5th vs MMP | Jan 2008-Sep 2013  Hot:95th vs MMP | Oct 2013-Dec 2015  Hot: 95th vs MMP |
| Non-accidental | Married | 504.75(241.06,768.43) | 302.32 (-163.44,768.08) | 21.37(-21.91,64.65) | 61.94 (-48.65,172.52) |
|  | <75 | 258.68(17.69, 499.68) | 148.78(-276.89, 574.45) | 16.35(-23.21,55.90) | 39.67(-61.40,140.73) |
|  | ≥75 | 246.06(152.51,339.61) | 153.54(-11.70,318.78) | 5.02(-10.34,20.38) | 22.27(-16.96,61.50) |
|  | Widowed | 365.64 (255.846,475.44) | -29.21(-223.14,164.73)* | 13.73(-4.30,31.75) | 21.64(-24.41,67.68) |
|  | <75 | 40.815(-19.77,101.40) | -78.51(-186.97,29.95) | 3.74(-6.23,13.71) | -6.64 (-32.60,19.31) |
|  | ≥75 | 246.06(152.51,339.61) | 76.59(-78.44,231.61)* | 8.45 (-5.95,22.86) | 29.29 (-7.51,66.10) |
| Cardiovascular | Married | 174.11(63.64,284.57) | 106.69 (-91.06,304.46) | -1.26 (-26.69,24.16) | 7.86 (-50.53,66.24) |
|  | <75 | 81.50(-13.64,176.64) | 46.71(-123.62,217.04) | -3.65(-25.55,18.25) | 9.45(-40.84,59.74) |
|  | ≥75 | 92.60(38.83,146.37) | 59.98(-36.28,156.24) | 2.39(-9.99,14.76) | -1.59(-30.01,26.83) |
|  | Widowed | 115.62 (53.76,177.48) | 23.80 (-86.95,134.55) | 3.21(-11.03,17.45) | -0.82 (-33.52,31.88) |
|  | <75 | 15.99(-16.10,48.09) | 12.91(-45.26,71.08) | -2.34(-9.75,5.07) | -5.69(-23.11,11.73) |
|  | ≥75 | 98.60(45.86,151.34) | 20.4(-74.03,114.83) | 5.11(-7.03,17.25) | 4.92(-22.96,32.79) |
| Respiratory | Married | 102.28 (44.59,159.97) | 61.65(-42.20,165.49) | 7.21 (-10.04,24.46) | 46.03(11.97,80.08)* |
|  | <75 | 24.63(-16.52,65.78) | 22.15(-51.92,96.22) | 2.02(-10.28,14.33) | 32.88(8.59,57.17)* |
|  | ≥75 | 77.65(39.48,115.82) | 39.50(-29.22,108.21) | 5.19(-6.22,16.60) | 13.14(-9.39,35.68) |
|  | Widowed | 132.90 (90.74,175.05) | 18.30 (-57.59,94.18)* | 9.10 (-3.50,21.71) | 21.21 (-3.67,46.10) |
|  | <75 | 24.63(-16.52,65.78) | 22.15(-51.92,96.22) | 1.62(-3.70,6.94) | 3.39(-7.43,14.21) |
|  | ≥75 | 123.92(85.51,162.32) | 24.00(-45.14,93.14)* | 7.51(-3.98,18.99) | 18.62(-4.05,41.30) |

MMP: minimum mortality percentile; The MMP for YLL of non-accidental, cardiovascular and respiratory mortality were 87, 83 and 79 percentiles, respectively. The 5th and 95th percentiles temperatures were 3.0℃and 30.4℃. **P*<0.05(comparison between two periods).

**Table S6. The cumulative cold and hot effects on mortality risk in the married and widowed people from 2008 to 2015 in Ningbo, China**

|  |  | Period | | Period | |
| --- | --- | --- | --- | --- | --- |
| Health Endpoints | Group | Jan 2008-Sep 2013  Cold: 5th vs MMP | Oct 2013-Dec 2015  Cold: 5th vs MMP | Jan 2008-Sep 2013  Hot: 95th vs MMP | Oct 2013-Dec 2015  Hot: 95th vs MMP |
| Non-accidental mortality | Married | 1.67 (1.38,2.03) | 1.42 (1.02,1.98) | 1.03 (0.99,1.08) | 1.05 (0.95,1.16) |
|  | <75 | 1.39(1.09,1.78) | 1.25(0.80,1.93) | 1.03(0.98,1.10) | 1.03(0.90,1.17) |
|  | ≥75 | 2.11(1.59,2.79) | 1.64(1.03,2.63) | 1.02(0.96,1.10) | 1.08(0.94,1.25) |
|  | Widowed | 2.36 (1.85,3.02) | 1.22 (0.79,1.89)* | 1.03 (0.97,1.09) | 1.10 (0.96,1.27) |
|  | <75 | 1.39(0.73,2.63) | 0.49(0.13,1.77) | 1.06(0.90,1.24) | 0.82(0.55,1.23) |
|  | ≥75 | 2.52(1.95,3.26) | 1.38(0.87,2.17)* | 1.02(0.96,1.09) | 1.14(0.98,1.31) |
| Cardiovascular mortality | Married | 1.81(1.27,2.57) | 1.49 (0.82,2.71) | 1.02 (0.93,1.11) | 1.02 (0.85,1.23) |
|  | <75 | 1.61(0.95,2.74) | 1.39(0.53,3.66) | 1.00(0.88,1.13) | 1.03(0.77,1.38) |
|  | ≥75 | 1.98(1.25,3.15) | 1.59(0.76,3.35) | 1.03(0.92,1.16) | 1.00(0.79,1.27) |
|  | Widowed | 1.98 (1.33,2.93) | 1.27 (0.65,2.47) | 1.04 (0.94,1.14) | 1.02 (0.83,1.26) |
|  | <75 | 1.79(0.49,6.47) | 1.86(0.14,25.36) | 0.85(0.61,1.17) | 0.60(0.25,1.46) |
|  | ≥75 | 1.99(1.31,3.00) | 1.27(0.64,2.52) | 1.05(0.95,1.16) | 1.05(0.85,1.31) |
| Respiratory mortality | Married | 2.48 (1.51,4.07) | 2.05 (0.83,5.03) | 1.03 (0.88,1.22) | 1.44 (1.05,1.98) |
|  | <75 | 1.55(0.55,4.36) | 2.69(0.37,19.57) | 1.03(0.73,1.45) | 2.22(1.10,4.49) |
|  | ≥75 | 2.82(1.63,4.88) | 1.95(0.73,5.21) | 1.04(0.87,1.24) | 1.30(0.92,1.83) |
|  | Widowed | 3.29 (2.13,5.10) | 1.71(0.74,3.95) | 1.13 (0.98,1.31) | 1.45(1.07,1.97) |
|  | <75 | 2.36(0.27,20.57) | 1.52(0.01,271.80) | 1.45(0.69,3.06) | 2.11(0.34,13.12) |
|  | ≥75 | 3.34(2.14,5.21) | 1.75(0.75,4.08) | 1.12(0.97,1.30) | 1.44(1.06,1.97) |

MMP: minimum mortality percentile; The MMP for RR of non-accidental, cardiovascular and respiratory mortality were 83, 83 and 79 percentiles, respectively. The 5th and 95th percentiles temperatures were 3.0℃and 30.4℃. **P*<0.05(comparison between two periods).

**
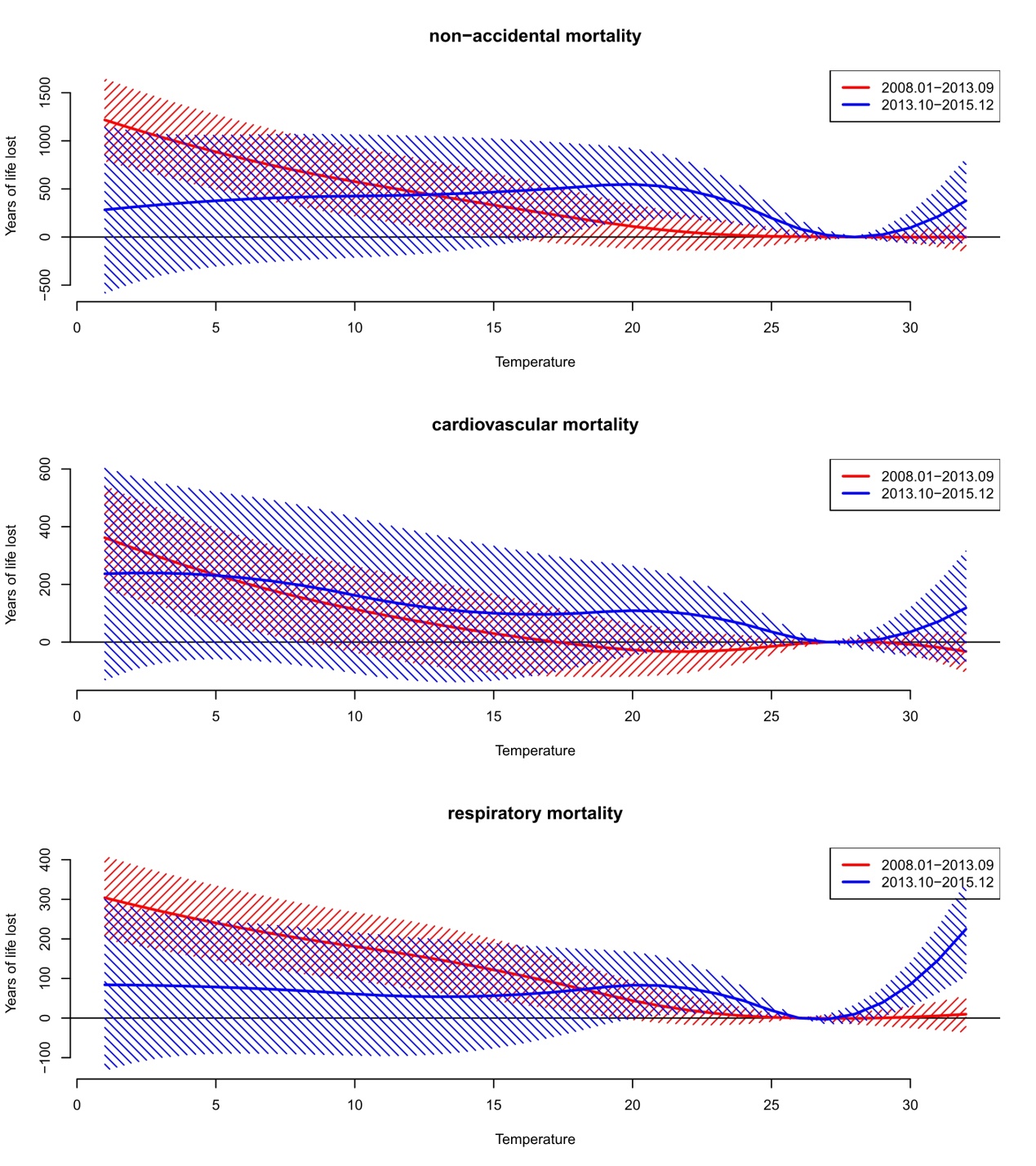
**

Fig.S1. Overall cumulative exposure-response curves (with 95% confidence intervals) for the association between temperature and YLL due to non-accidental, cardiovascular and respiratory mortality during period I (Jan 2008 to Sep 2013, red) and period II (Oct 2013 to Dec 2015, blue) when extending the maximum lag period to 27 days.

**
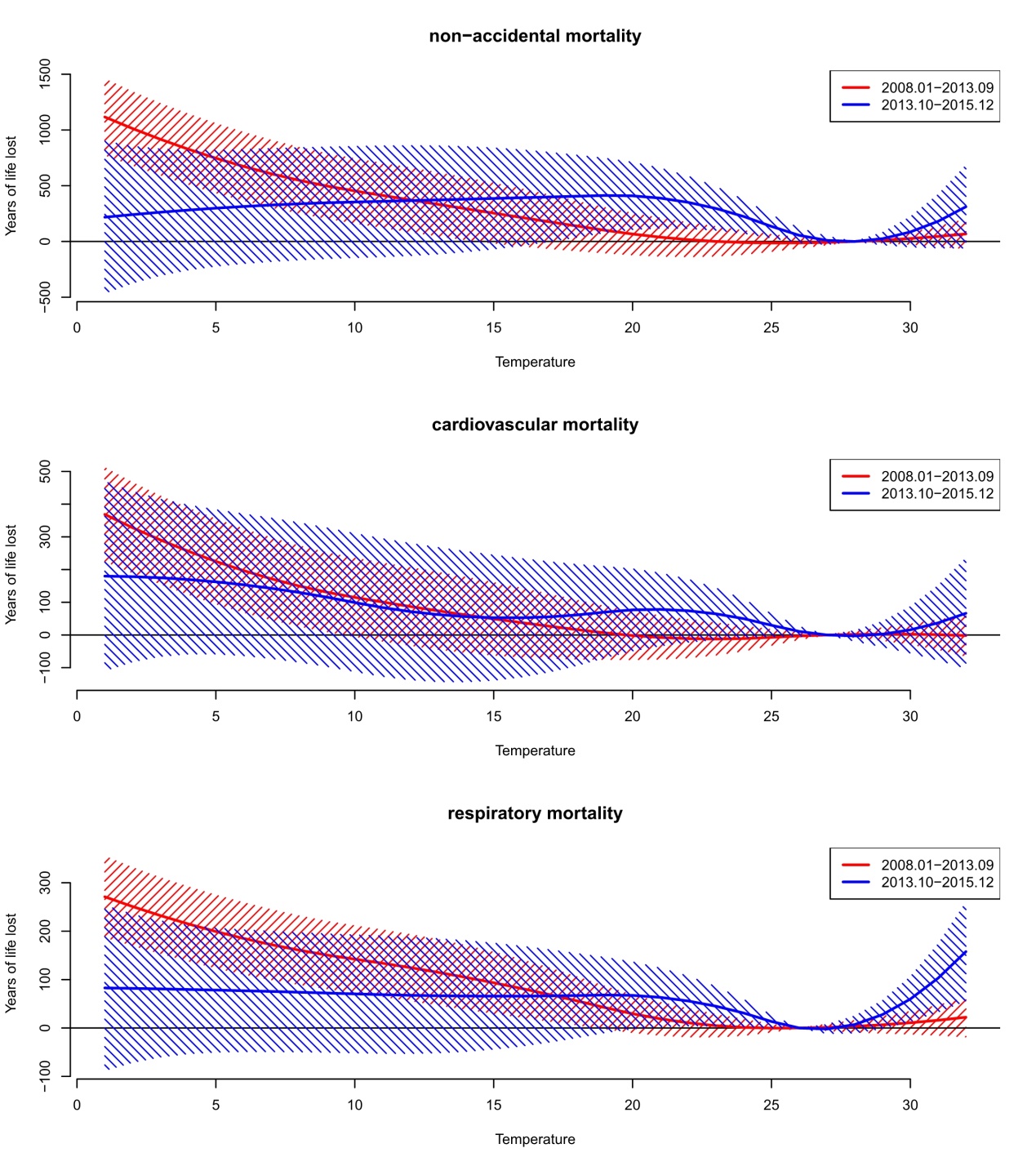
**

Fig.S2. Overall cumulative exposure-response curves (with 95% confidence intervals) for the association between temperature and YLL due to non-accidental, cardiovascular and respiratory mortality during period I (Jan 2008 to Sep 2013, red) and period II (Oct 2013 to Dec 2015, blue) when using 6 degrees of freedom per year for the time variable.


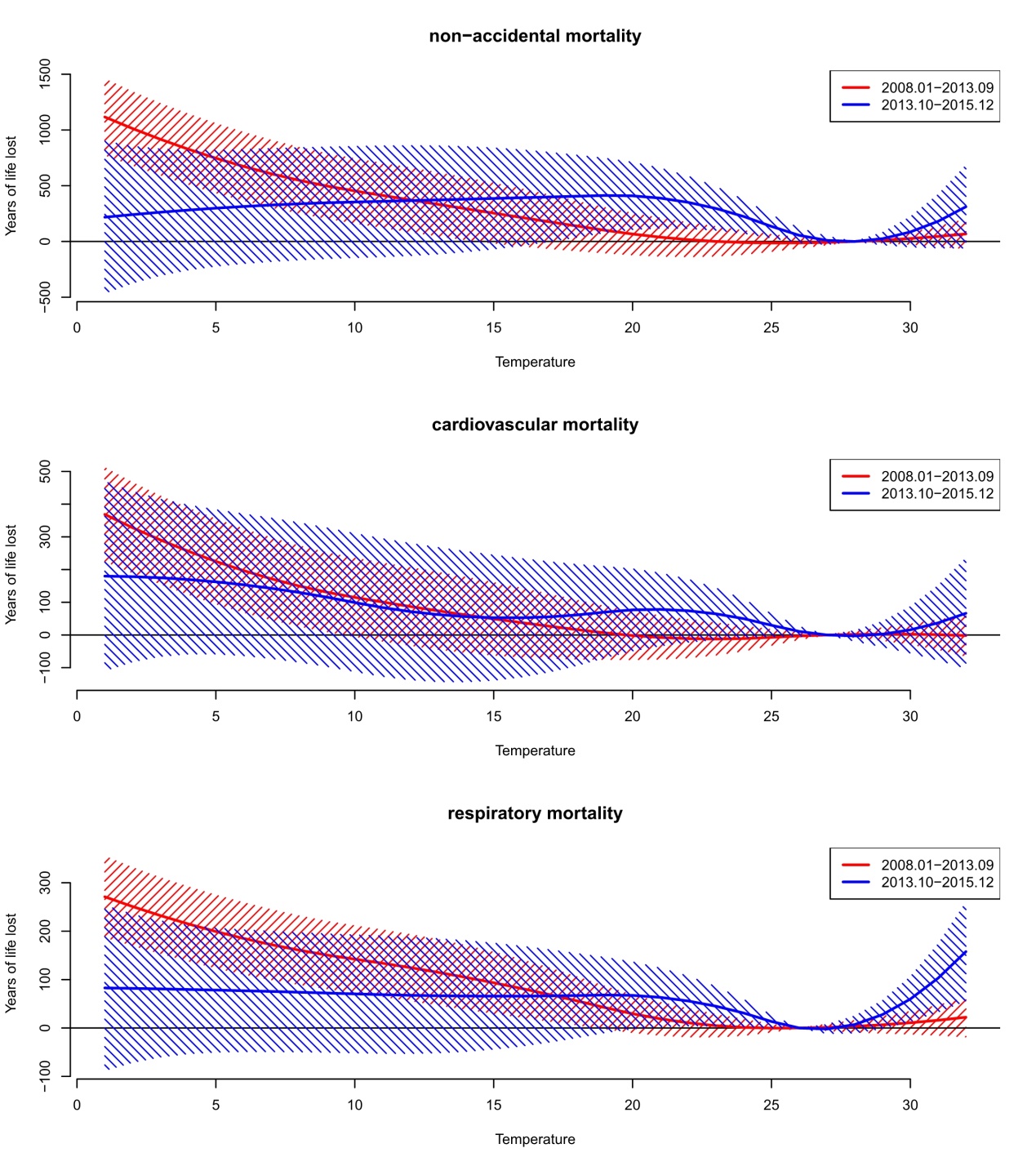


Fig.S3. Overall cumulative exposure-response curves (with 95% confidence intervals) for the association between temperature and YLL due to non-accidental, cardiovascular and respiratory mortality during period I (Jan 2008 to Sep 2013, red) and period II (Oct 2013 to Dec 2015, blue) when using 8 degrees of freedom per year for the time variable.

**
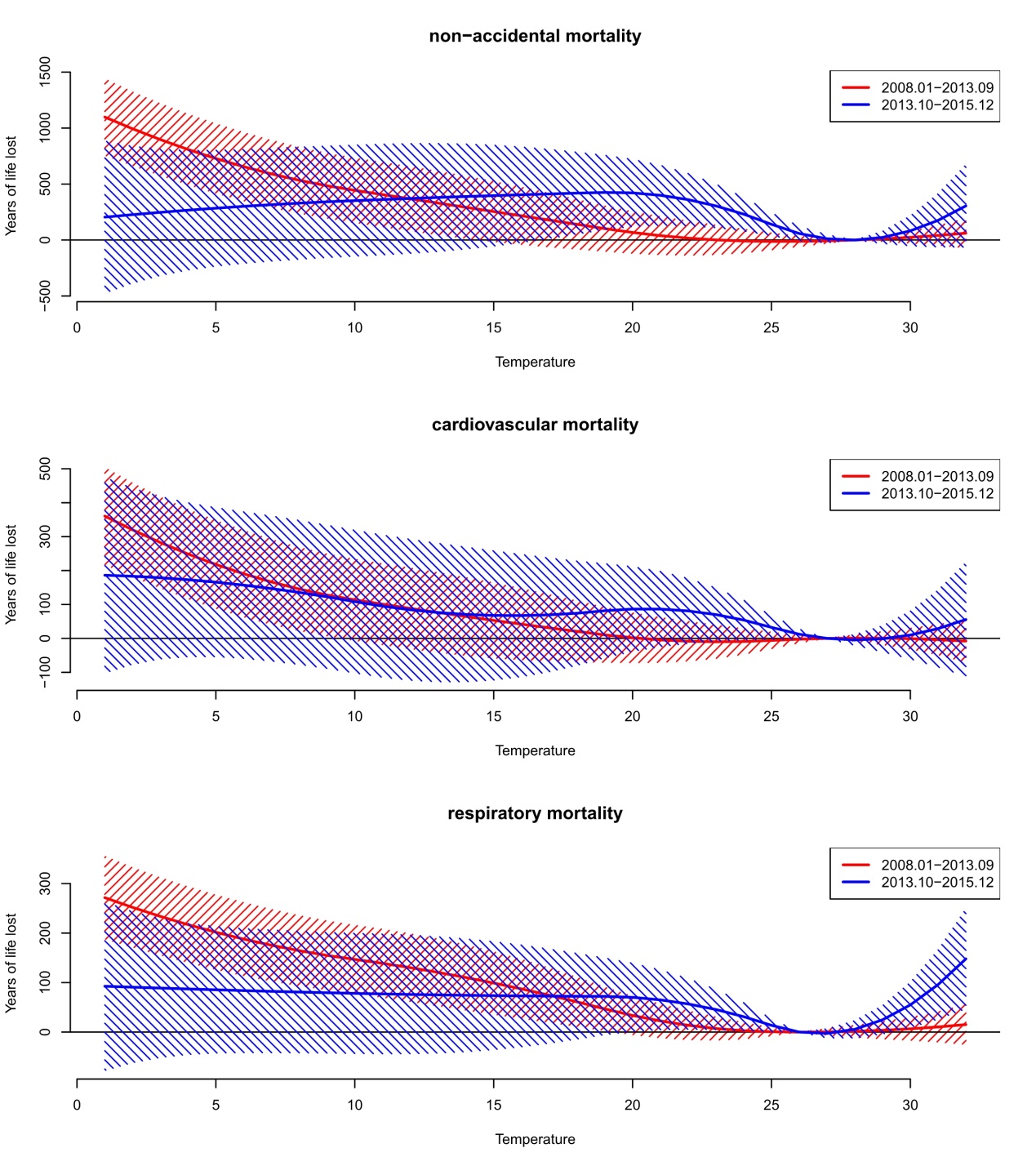
**

Fig.S4. Overall cumulative exposure-response curves (with 95% confidence intervals) for the association between temperature and YLL due to non-accidental, cardiovascular and respiratory mortality during period I (Jan 2008 to Sep 2013, red) and period II (Oct 2013 to Dec 2015, blue) when removing relative humidity from the analysis.

**
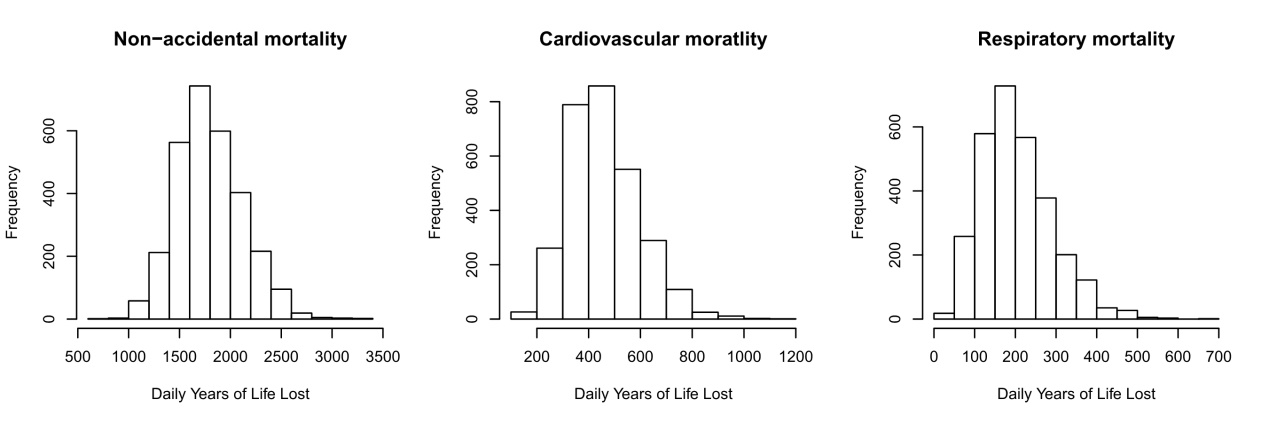
**

Fig.S5. Distribution of daily YLL for non-accidental, cardiovascular and respiratory mortality.
